# Supplementary material for: Mass media pressure on physical build, psychological well-being and physical-healthy profile. An explanatory model in adulthood
Source: PeerJ. 2023 Jan 11;11:e14652. doi: 10.7717/peerj.14652 (PMC9840389; doi:10.7717/peerj.14652)
Supplement: Supplemental Information 2 [file peerj-11-14652-s002.docx]

The questionnaires have been obtained from different doctoral theses.

The questionnaires SATAQ-4 and PWBS have been obtained from the following doctoral thesis: <https://digibug.ugr.es/handle/10481/63369>

Likewise, the PREDIMED questionnaire has been obtained from the following link: <http://www.predimed.es/uploads/8/0/5/1/8051451/p14_medas.pdf>

In this case, the documents consulted are fully accessible to the entire population, following a CC BY-NC-ND 3.0 ES licence, which allows the material to be shared, copied and redistributed in any medium or format.
